# Prospective Epigenetic Actions of Organo-Sulfur Compounds against Cancer: Perspectives and Molecular Mechanisms

**DOI:** 10.3390/cancers15030697

**Published:** 2023-01-23

**Authors:** Shoaib Shoaib, Mohammad Azam Ansari, Mohammed Ghazwani, Umme Hani, Yahya F. Jamous, Zahraa Alali, Shadma Wahab, Wasim Ahmad, Sydney A. Weir, Mohammad N. Alomary, Nabiha Yusuf, Najmul Islam

**Affiliations:** 1 Department of Biochemistry, Faculty of Medicine, Aligarh Muslim University, Aligarh 202001, Uttar Pradesh, India; 2 Department of Epidemic Disease Research, Institute for Research and Medical Consultations (IRMC), Imam Abdulrahman Bin Faisal University, Dammam 31441, Saudi Arabia; 3 Department of Pharmaceutics, College of Pharmacy, King Khalid University, Abha 62529, Saudi Arabia; 4 Vaccine and Bioprocessing Center, King Abdulaziz City for Science and Technology (KACST), Riyadh 11442, Saudi Arabia; 5 Department of Clinical Laboratory Sciences, College of Applied Medical Sciences, University of Hafr Al Batin, Hafr Al Batin 31991, Saudi Arabia; 6 Department of Pharmacognosy, College of Pharmacy, King Khalid University, Abha 61421, Saudi Arabia; 7 Department of Pharmacy, Mohammed Al-Mana College for Medical Sciences, Dammam 34222, Saudi Arabia; 8 Department of Dermatology, University of Alabama at Birmingham, Birmingham, AL 35294, USA; 9 National Centre for Biotechnology, King Abdulaziz City for Science and Technology (KACST), Riyadh 11442, Saudi Arabia

**Keywords:** cancer, epigenetics, organo-sulfur compounds, apoptosis, cell cycle

## Abstract

**Simple Summary:**

Epigenetics has provided a new dimension to our understanding of cancer initiation and progression. Unlike the genetic changes in DNA sequence, abnormal epigenetic alterations may also lead to cancer development. Aberrant modulations in the functions of DNA methyltransferases (DNMTs) and histone deacetylases (HDACs) are the fundamental events of the epigenome and contribute significantly to the pathogenesis of cancer. Interestingly, antioxidant, anti-proliferative, anti-inflammatory, anti-metastasis, anti-malarial, antiviral, antibacterial, and antifungal properties of plant-derived bioactive compounds have been extensively researched in recent decades. According to the American Cancer Society, there will be a 23.6 million increase in new cancer cases by 2030. Due to the severity of the increased risk, it becomes very important to develop novel drug candidates from natural sources and evaluate various preventive measures against cancer. Numerous studies have indicated that OSCs can target epigenetic mechanisms, representing an enticing strategy for therapeutic intervention. These OSCs, when administered in a dose-dependent and combinatorial-based manner, can have an enhanced impact on epigenetic alterations, which may lead to the cancer prevention and therapy.

**Abstract:**

Major epigenetic alterations, such as chromatin modifications, DNA methylation, and miRNA regulation, have gained greater attention and play significant roles in oncogenesis, representing a new paradigm in our understanding of cancer susceptibility. These epigenetic changes, particularly aberrant promoter hypermethylation, abnormal histone acetylation, and miRNA dysregulation, represent a set of epigenetic patterns that contribute to inappropriate gene silencing at every stage of cancer progression. Notably, the cancer epigenome possesses various HDACs and DNMTs, which participate in the histone modifications and DNA methylation. As a result, there is an unmet need for developing the epigenetic inhibitors against HDACs and DNMTs for cancer therapy. To date, several epigenetically active synthetic inhibitors of DNA methyltransferases and histone deacetylases have been developed. However, a growing body of research reports that most of these synthetic inhibitors have significant side effects and a narrow window of specificity for cancer cells. Targeting tumor epigenetics with phytocompounds that have the capacity to modulate abnormal DNA methylation, histone acetylation, and miRNAs expression is one of the evolving strategies for cancer prevention. Encouragingly, there are many bioactive phytochemicals, including organo-sulfur compounds that have been shown to alter the expression of key tumor suppressor genes, oncogenes, and oncogenic miRNAs through modulation of DNA methylation and histones in cancer. In addition to vitamins and microelements, dietary phytochemicals such as sulforaphane, PEITC, BITC, DADS, and allicin are among a growing list of naturally occurring anticancer agents that have been studied as an alternative strategy for cancer treatment and prevention. Moreover, these bioactive organo-sulfur compounds, either alone or in combination with other standard cancer drugs or phytochemicals, showed promising results against many cancers. Here, we particularly summarize and focus on the impact of specific organo-sulfur compounds on DNA methylation and histone modifications through targeting the expression of different DNMTs and HDACs that are of particular interest in cancer therapy and prevention.

## 1. Introduction

Cancer is a state of hyper-proliferation characterized by the development of abnormal clones that divide uncontrollably and invade surrounding tissues by acquiring metastases. Globally, cancer is the second-leading cause of death, and every year, millions of people are diagnosed with it. According to global cancer statistics, 19.3 million new cases and approximately 10 million deaths were reported in 2020 [1]. Despite technological advances and improved treatment strategies, its mortality and incidence remain high, particularly in low- and middle-income countries, and cancer poses a huge challenge to all nations [2]. Cancer development is a multistage and multifactorial process, with stages I (early-stage cancer), II and III (cancer cell invasion into nearby tissues or lymph nodes), and IV (cancer cells with metastatic properties) being the most common. Generally, blood tests, immunohistochemistry, and imaging techniques such as computed tomography (CT), magnetic resonance imaging (MRI), and positron emission tomography (PET) scanning are performed to diagnose a cancer type in clinical oncology [3]. Numerous risk factors have been recognized as major causes of cancer, contributing to the regional and global burden of the disease. For example, smoking [4], alcohol [5], obesity [6], genetic predisposition [7], some viral infections [8], exposure to cancer-causing chemicals [9], and chronic inflammation [10] are the most studied risk factors in cancer. Currently, cancer treatment mainly relies on conventional approaches, including surgery, radiotherapy, chemotherapy, immunotherapy, and chemoradiotherapy as a combined treatment strategy. However, each of these treatments may have low-to-serious side effects. Chemotherapy, in particular, is associated with mild, moderate, and severe side effects affecting the skin, hair, blood, kidneys, and gastrointestinal tract, as well as hepatotoxicity, neurotoxicity, cardiotoxicity, ototoxicity, and other conditions [11].

Importantly, cancer is characterized by uncontrolled cell proliferation, cell migration, invasion, hypoxic conditions, angiogenesis, and vascular functions. Moreover, there are several cancer markers, including epidermal growth factor receptor (EGFR), vascular endothelial growth factor (VEGF), αvβ3 integrin, carcinoembryonic antigen (CEA), folate receptors, transferrin receptors, somatostatin receptors, and prostate stimulating membrane antigen (PSMA) [12]. Extensive research in the last few decades has revealed that cancer develops as a consequence of dysregulation of several signaling pathways leading to increased expression of EGFR, cyclooxygenase-2 (COX-2), nuclear factor (NF)-κB, interleukin (IL)-6, and IL-1, prostaglandins, cyclin D1 and cyclin E (cell cycle proteins), vascular endothelial growth factor (VEGF), matrix metalloproteinases (invasion-promoting proteins), Bcl-2, Bcl-X_L_, survivin, and XIAP (cell survival proteins), cMyc, and AP-1 (cellular proliferation) [13]. Inflammation has long been correlated with the development of cancer due to the involvement of chronic inflammatory mediators, which are known to exert pleiotropic effects in the development of cancer. For instance, cancer-associated inflammation signaling favors tumor growth, proliferation, migration, invasion, and malignant transformation, which are achieved by up-regulation of hypoxia inducible factor-1 (HIF-1), signal transducer and activator of transcription 3 (STAT-3), NF-κB, IL-6, IL-1, and tumor necrosis factor (TNF) [14]. In line with these observations, previous studies indicate that apart from genomic instability, inflammation remains a potential risk factor for cancer development because of its ability to enable escape from immune surveillance, apoptosis inhibition, enhanced vascularization, and metastasis [15]. In the early stages of cancer, inflammation-inducing molecular mediators such as reactive oxygen and nitrogen species and cytokines from tumor-infiltrating immune cells modulate epigenetic regulation, leading to the suppression of tumor suppressor gene activation [12]. Tissue transglutaminase (TG2) significantly induces epithelial-mesenchymal transition (EMT), which enhances tumor invasiveness and metastatic properties in conjunction with TGF- and NF-κB signaling, indicating a strong correlation between TG2, inflammation, and cancer [16,17].

**Figure 1:**
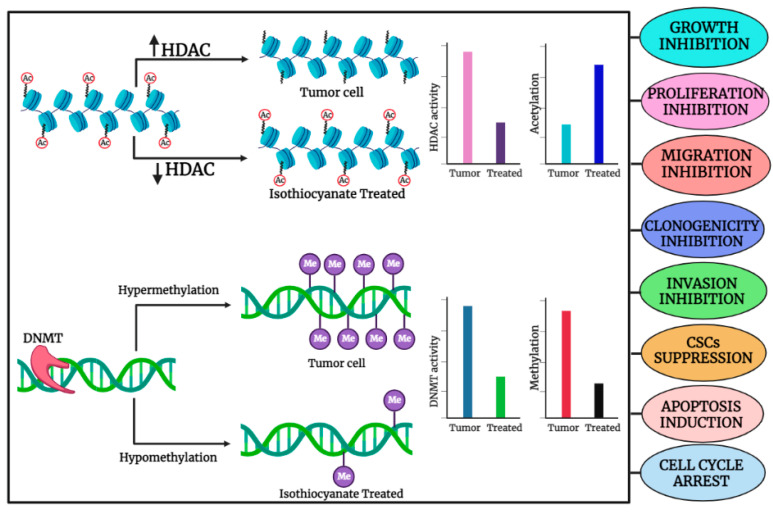
General illustration depicting the role of organo-sulfur compounds in the regulation of HDAC and HDAC activities and their impact on methylation and acetylation, respectively, in cancer cell. The image was created in online software, Biorender.

Epigenetic machinery is composed of DNA methylation and histone modifications that significantly impact gene expression. Gene transcription involves histone acetylation induced by histone acetyl transferases, while gene silencing includes histone hypoacetylation mediated through histone deacetylases. Aberrant methylation often results in cancer development through the many-fold expression of DNA methyltransferases (DNMT1, DNMT3a, and DNMT3b) that is comparatively significantly higher than their normal expression in healthy cells [18]. Epigenetic alterations in regulatory genes encoding histone deacetylases (HDACs) and DNMTs have been correlated with several cancer-related cellular processes, including abnormal cell proliferation, metastasis, cell cycle dysregulation, and evasion of apoptosis [19]. Emerging data on molecular mechanisms involved in cancer indicate that several microRNAs actively contribute to the initiation and progression of the disease. Additionally, histone modification, DNA methylation, nucleosome positioning, and non-coding RNAs control the epigenetic regulation of cancer cells that can lead to the initiation and progression of cancer. Apart from mutational events, epigenetic mechanisms are considered alternative methods to disrupt the function of tumor suppressor genes contributing to tumorigenesis. Aberrant promoter CpG island hypermethylation and transcriptional silencing of pro-differentiation factors and tumor suppressor genes are both associated with the initiation and progression of cancer [20]. An imbalance between cell division and apoptosis is an important factor that may foster the survival, transformation, and progression of cancer cells through various epigenetic mechanisms. Importantly, modulation of apoptosis-associated genes induces tumorigenic and metastatic progression of cancer cells by epigenetic silencing of occludin, a tight junction protein [21]. In almost every cancer, promoter methylation is one of the key mechanisms of gene silencing. Several studies have demonstrated that hypermethylation of the promoter region of different genes has a strong association with cancer. SMAD14 down-regulation is often correlated with gastric cancer metastasis and is attributed to the hypermethylation of the SMAD14 promoter region [22]. A general illustration of the role of organo-sulfur compounds in the regulation of HDAC and HDAC activities, as well as their effects on methylation and acetylation in cancer cells, is shown in Figure 1.

Cell adhesion and recognition are mediated by H-cadherin (CDH13), and one of the interesting in vitro studies showed decreased expression of CDH13 in B cell lymphomas, which was owed to aberrant methylation on the promoter region of CDH13 [23]. Another important clinical study demonstrated hypermethylation of RUNX family transcription factor 3 (RUNX3) in the serum samples of gastric, non-small cell lung cancer (NSCLC), and pancreatic, liver, and colorectal cancers, whereas no methylation was detected in healthy samples [24]. S100A6 and S100A2 are the two calcium-binding proteins that are mostly down-regulated in prostate cancer, and the underlying mechanism responsible for it appears to involve hypermethylation of S100A6 and S100A2 [25]. Previous in vitro research on head and neck cancer found 16.6% cytosine methylation in the CpG dinucleotides of the retinoblastoma 1 (RB1) gene, which is an important cell cycle regulator [26]. Solid and hematopoietic malignancies are characterized by aberrant SOX11 expression, which serves as a prognostic marker. Furthermore, hypermethylation of the SOX11 promoter has been reported in gastric cancer and has been linked to a poor prognosis [27]. Retinoblastoma-protein-interacting zinc finger (RIZ1) is a tumor suppressor gene, and its hyper-methylation was linked with the early tumorigenesis of hepatocellular carcinoma [28]. Aberrant methylation of Adenomatous Polyposis Coli (APC), Dickkopf (DKK), axis inhibition protein 2 (AXIN2), and secreted frizzled-related protein (SFRP) has been identified in colorectal cancer [29,30,31]. A study on colorectal cancer showed that DNMT1 overexpression is associated with promoter methylation of WNT pathway regulators that include WNT inhibitory factor-1 (WIF1), adenomatous polyposis coli (APC), and Dickkopf-3 (DKK3) [32].

## 2. Organo-Sulfur Compounds and Their Epigenetic Modulatory Actions against Cancer

**Figure 2:**
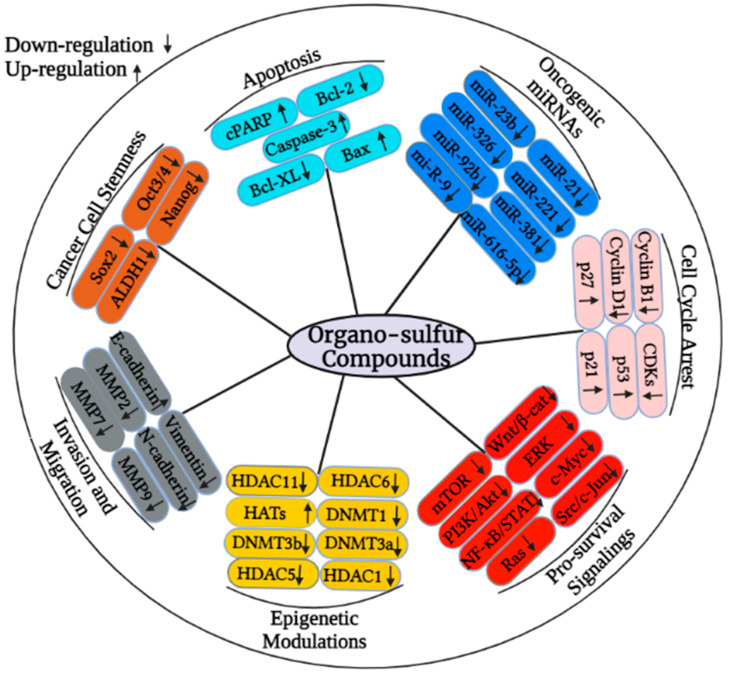
Organo-sulfur compounds can change the expression of various cancer-associated proteins though the modulating of epigenetic mechanisms. In particular, organo-sulfur compounds may target the activity of HDACs and DNMTs to suppress the proliferation-, metastasis-, cell cycle progression, and pro-survival-associated signaling molecules. These compounds can also target the regulation of micro RNAs to restrict the cancer growth.

Organo-sulfur compounds are bioactive dietary compounds that are mainly obtained from cruciferous vegetables such as broccoli, cabbage, cauliflower, watercress, and Brussels sprouts. Notably, organo-sulfur compounds such as sulforaphane (SFN), phenethyl isothiocyanate (PEITC), allicin, benzyl isothiocyanate (BITC), and diallyl disulfide (DADS) are naturally occurring small chemical entities that are derived from the glucosinolate precursors found in cruciferous plants. Epidemiological data show that consumption of cruciferous vegetables may lead to a decreased overall risk of many diseases, including different types of cancer. Organo-sulfur compounds have previously been shown to have chemopreventive effects on a variety of cancers via epigenetic modulation. This review discusses established anticancer properties, with a special emphasis on epigenetic regulatory mechanisms to target cancer growth, proliferation, metastasis, cell cycle, and apoptosis regulation through organo-sulfur compounds as depicted in Figure 2. Organo-sulfur compounds selectively target the down-regulation of several proteins that contribute to cancer initiation and progression while up-regulating proapoptotic proteins, tumor suppressors, and cell cycle arresting molecules, as shown in Figure 2.

**Figure 3:**
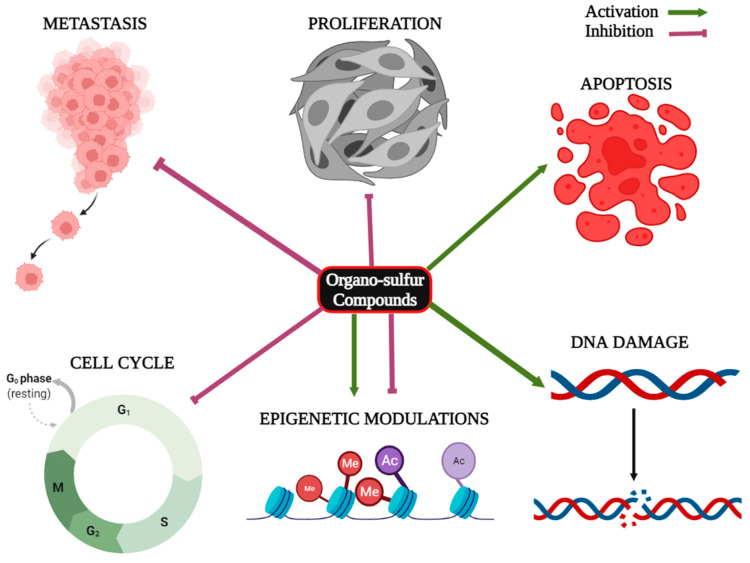
Role of organo-sulfur compounds in the inhibition of proliferation, metastasis and cell cycle progression, apoptosis induction, DNA damage, and epigenetic modulations in cancer.

Micro RNAs (miRNAs) are small, evolutionarily conserved, non-coding endogenous single-stranded RNA molecules consisting of 18–25 nucleotides that participate in the post-transcriptional regulation of gene expression. Numerous studies have confirmed that dysregulation of miRNAs actively contributes to the development, progression, and metastasis of cancer [33]. Organo-sulfur compounds have been documented to exert anticancer effects through the modulation of the expression of different cancer-associated miRNAs [34]. Moreover, studies suggest that organo-sulfur compounds can affect the expression of oncogenes, tumor suppressor genes, and various cancer-associated proteins. The anti-proliferative effects of organo-sulfur compounds have been summarized in Table 1. Figure 3 is showing the effects of organo-sulfur compounds against cancer. 

### 2.1. Sulforaphane (SFN)

Numerous preclinical as well as clinical studies have shown the pharmacological importance of sulforaphane (SFN) against several infectious and non-infectious diseases. Particularly, SFN’s ability to act as an antioxidant and anti-inflammatory agent is supposed to be responsible for its chemopreventive and chemotherapeutic role against ovarian cancer, cervical cancer, breast cancer, pancreatic cancer, prostate cancer, nasopharyngeal cancer, melanoma, liver cancer, and lung cancer. Dietary phytocompounds such as curcumin, resveratrol, epigallocatechin gallate (EGCG), and sulforaphane have been documented to modulate the epigenetic mechanisms of cancer, which may be due to alterations in histone acetylation and methylation at the promoter region of many oncogenic genes. SFN has previously been shown to have anti-proliferative effects against breast cancer via increased expression of p53, p21, and the phosphatase and tensin homolog (PTEN), which was associated with epigenetic modulation of DNMT1. Furthermore, SFN-treated breast cancer cells (MCF-7 and MDA-MB-231) demonstrated decreased methylation of PTEN and RARβ2 promoters [35]. In vitro studies demonstrated that SFN significantly suppressed the growth and proliferation of MCF-7 and MDA-MB-231 cells. SFN treatment inhibited telomerase reverse transcriptase (hTERT) activity, leading to apoptosis induction in hTERT down-regulated breast cancer cells, and it was suggested that SFN down-regulatedDNMT1 and DNMT3 [36]. The anti-proliferative effects of SFN on nasopharyngeal carcinoma were investigated, and the results of the study showed that SFN inhibited the formation of nasopharyngeal carcinoma tumor spheres and reverted expression of WIF1 along with the down-regulation of DNMT1 [37]. An investigation into prostate cancer prevention has highlighted one of the remarkable mechanisms that contribute to the anti-proliferative effects of SFN. The study showed that SFN significantly promoted demethylation of the cyclin D2 promoter that contains cMyc and Sp1 binding sites, which was associated with a lower expression of DNMT1 and DNMT3b [38].

Furthermore, SFN has the potential to modulate abnormal epigenetic mechanisms. In an in vitro study, SFN-treated HepG2 liver cancer cells showed increased proliferation inhibition and apoptosis induction, which could be due to the DNA damage and mitotic spindle abnormalities. The mechanistic insights suggest that SFN treatment inhibited the activities of HDAC5 and HDAC11 as well as SFN-mediated hypermethylation of E2F3 and Thanatos-associated (THAP) domain-containing apoptosis-associated protein 1 (THAP1) and hypomethylation of cytoplasmic polyadenylation element-bindingprotein-2 (CPEB2) [39]. A growing body of interest indicates that epigenetic alterations are prominently involved in cancer initiation and progression. SFN-treated TRAMP C1 cells showed de-methylation of CpG dinucleotides in the promoter region of nuclear factor erythroid 2–related factor 2 (Nrf2) and attenuated the expression of DNMT1 and DNMT3a. SFN-treated TRAMP C1 cells showed increased activity of -Histone 3, Nrf2, and NAD(P)H Quinone Dehydrogenase 1 (NQO-1), and a reduced expression of HDACs was also reported in SFN-treated cells [40]. Cancer cells are marked by the down-regulation of tumor suppressor genes and up-regulation of oncogenes. Therefore, in the last few decades, the researchers have been engaged to identify natural compounds that can restore the function of tumor suppressor genes as well as target the suppression of oncogenic signaling pathways. One such effort led to the identification of SFN as an anti-proliferative agent against cervical cancer, in which SFN-exposed cervical cancer cells restored the expression of cadherin 1 (CDH1), death-associated protein kinase 1 (DAPK1), retinoic acid receptor (RARβ), and glutathione S-transferase Pi 1 (GSTP1), which was possibly due to the reduction in the expression of HDAC1 and DNMT3b [41]. SFN exposure to skin cancer cells resulted in the inhibition of cellular transformation and enhanced expression of Nrf2, NQO-1, and HO-1. The mechanistic insights revealed a decreased methylation ratio of CpG dinucleotides in the promoter region of Nrf2 in SFN-treated skin cancer cells, and SFN also attenuated the expression of HDACs (HDAC1, HDAC2, HDAC3, and HDAC4) and DNMTs (DNMT1, DNMT3a, and DNMT3b) [42].

Moreover, an in vitro study demonstrated that SFN inhibited the expression and activity of hTERT in prostate cancer cell lines, which was linked with the acetylation of histone H3 lysine 18 and the di-methylation of histone H3 lysine 4 (H3K4) [43]. Another study showed that SFN and iberin have the potential to modulate histone acetylation in malignant melanoma cells. The study further suggested that SFN and iberin reduced the viability of skin cancer cells (A375, Hs294T, and B16F10) and decreased the expression of HDACs (HDAC1, HDAC2, HDAC4, and HDAC6). SFN- and iberin-treated A375 cells also showed an increased activity of histone acetyl transferase and reduced expression of CBP [82]. Intriguingly, SFN administration to breast cancer cells led to the down-regulation of HDAC5 through the repression of the upstream stimulatory factor-1 (USF1) activity. Furthermore, SFN treatment enhanced lysine-specific histone demethylase 1A (LSD1) ubiquitination and degradation in MDA-MB-231-induced xenograft tumors, suggesting the HDAC5-LSD1 axis as a putative drug target [44]. Expending data on chemoprevention by organo-sulfur compounds shows that SFN exerted anti-proliferative effects on lung cancer cell lines (A549 and H1299) through apoptosis induction and cell cycle arrest at the S phase, whereas an in vivo study showed that SFN administration inhibited tumor growth in a mouse model. The mechanistic insights revealed that SFN suppressed HDAC expression and enhanced the acetylation of H3 and H4 histones in both lung cancer cell lines [45]. Another in vivo study on colon cancer indicated that SFN treatment significantly reduced the tumor burden in Nrf^−/+^-deficient mice. In particular, SFN-treated tissue lysates from colon tumors displayed decreased HDAC3 levels and CDKN2a/p16 mRNA expression and enhanced global histone H4 acetylation [46]. A study revealed that SFN treatment caused G_2_/M arrests during which normal control cells showed increased HDAC3 activity but not in SFN-treated colon cancer cells. Furthermore, SFN treatment resulted in the phosphorylation of silencing mediators for retinoid and thyroid hormone receptors (SMRT) and caused dissociation of HDAC3/SMRT while increasing the HDAC3 binding with peptidyl-prolyl cis/trans isomerase 1 (Pin1) [47].

Overexpression of miRNA-9 and miRNA-326 is correlated with gastric cancer carcinogenesis, which targets caudal type homeobox 1 (CDX1) and CDX2 (tumor suppressors) by promoting hypermethylation of the promoter region of CDX1 and CDX2 [83]. Treating gastric cancer cell lines (AGS and MKN4) with SFN led to the decreased proliferation, apoptosis induction, and reduced expression of miRNA-9 and miRNA-326, and thereby, SFN enhanced levels of CDX1 and CDX2 in AGS and MKN4 [48]. Hypermethylation of the promoter region of miR-9-3 led to the down-regulation of miR-9-3 in lung cancer. SFN treatment on to A549 lung cancer cells resulted in the increased miR-9-3 expression that could be due to the reduction in the methylation of the promoter of miR-9-3. SFN also attenuated the expression of cadherin 1, DNMT3a, HDAC1, HDAC3, and HDAC6 in A549 cells [49]. Moreover, SFN-exposed MCF-7, MDA-MB-231, and SK-BR-3 breast cancer cells underwent cell cycle arrest through the augmented levels of p21 and p27 and apoptosis induction. The mechanistic insights revealed the down-regulation of protein kinase B (Akt) signaling, AMP-activated protein kinase (AMPK) activation, as well as global DNA hypomethylation, and reduced levels of DNMT1 and DNMT3b that could be possibly due to reduced levels of miR-23b, miR-92b, miR-381, and miR-382 in response to SFN treatment [50]. SFN inhibited proliferation and induced apoptotic cell death in HCT 116 and RKO cells, whereas G2/M cell cycle arrest was reported only in RKO cells. Upon exploring the molecular mechanism of SFN, consistent down-regulation of hTERT and HDAC1 was reported, which was further backed up by suppressed expression of oncogene miR-21 [51]. 

Several miRNAs are known to act as onco-miRNAs, which are overexpressed in lung cancer tissues. Specifically, up-regulation of miR-616-5p is associated with the development and progression of non-small cell lung cancer (NSCLC). Therefore, in a study, SFN-exposed non-small cell lung cancer cells were shown to inhibit proliferation, migration, invasion, and epithelial to mesenchymal transition in 95D and H1299 lung cancer cells. Interestingly, the anti-metastatic effects of SFN on NSCLC cells occurred as a consequence of down-regulation of miR-616-5p, which could lead to enhanced E-cadherin expression and decreased expression of vimentin, N-cadherin, and β-catenin through the impairment of GSK3β levels [52]. In addition to the suppression of cancer cell stems through ALDH1 inhibition, SFN-treated oral squamous cell carcinoma cells also showed significant inhibition of proliferation, migration, invasion, clonogenicity, and in vivo tumorigenic potential in a xenograft model, which was further correlated with the increased expression of miR-200c [53]. Further investigations into SFN-mediated anticancer effects indicate that treating bladder cancer T24 cells with SFN resulted in the suppression of cell migration, invasion ability, and the inhibition of the epithelial to mesenchymal transition. Upon exploring the molecular mechanism of SFN-mediated anti-metastatic effects, it was reported that SFN reduced expression of Zinc finger E-box-binding homeobox 1 (ZEB1), COX-2, MMP-2, MMP-9, and Snail owing to increased expression of miR-200c in T24 cells [54]. Micro RNA-19 is a prominent oncogenic miRNA that contributes to the regulation of Wnt/β-catenin oncogenic signaling. Previously, miR-19a and miR-19b were reported to be up-regulated by cancer stem cells (CSCs) that could promote tumorsphere formation by Wnt/β-catenin pathway activation and β-catenin/T-cell factor/lymphoid enhancer factor (TCF) transcriptional activity in lung cancer cells. SFN treatment suppressed the lung cancer CSCs by down-regulating miR-19 and the inhibition of Wnt/β-catenin pathway activation [55]. SFN inhibited CSCs proliferation and tumorsphere formation and induced apoptosis in HONE1 and SUN1 nasopharyngeal cancer cells, which could be due to the attenuation of the expression of c-Myc, Oct-3/4, Sox-2, Nanog, and β-catenin. SFN-mediated inhibition of CSC markers was due to the decreased expression of STAT3 by the up-regulation of miR-124-3p in HONE1 and SUN1 cells [56].

### 2.2. Phenethyl Isothiocyanate (PEITC)

Ras-association domain family 1 isoform A (RASSF1A) is a tumor suppressor gene, and hypermethylation of the promoter region of RASSF1A has been linked to the development of many cancers. An interesting study showed that PEITC induced apoptosis in LNCaP prostate cancer cells through the increased expression of Bax and caspase-3 and arrested cell cycle progression by down-regulating cyclin B1 as well as enhanced p21 activity. Mechanistic insights revealed that PEITC down-regulated the expression of DNMT3a, DNMT3b, HDAC1, HDAC2, HDAC4, and HDAC6 through the increased de-methylation of the RASSF1A promoter, which led to apoptosis induction and cell cycle arrest through the reactivation of RASSF1A [57]. Previous research has shown that CDH1 silencing is a major risk factor in the development and progression of breast cancer, which is accomplished by fostering the hypermethylation of its promoter region. PEITC-treated breast cancer cells displayed the reactivation of CDH1 that could be due to the decreased activity of DNMT1, DNMT3a, DNMT3b, HDAC1, and HDAC2. Notably, PEITC inhibited colony and mamosphere formation by decreasing the expression of c-Myc, ALDH-1, Oct-4A, and SOX2, which are key regulatory proteins for CSCs [58]. In cancer, the polycomb group (PcG) is frequently hyperactivated. PEITC exposure to colon cancer cells led to hypomethylation of PcG and blocked HDAC binding to euchromatin, which was linked to reduced tumor growth, apoptosis induction, and cell cycle arrest. Furthermore, PEITC treatment significantly enhanced mRNA expression of pro-apoptotic proteins and attenuated activator protein 1 (AP1) and NF-κB expression. PEITC also down-regulated PcG complex proteins, including B cell-specific Moloney murine leukemia virus integration site 1 (BMI1), enhancer of zeste homolog 2 (EZH2), and suppressor of zeste 12 homolog (SUZ12), whereas long-term PEITC exposure to colon cancer cells caused the hypomethylation of PcG target genes such as Protocadherin 10 (PCDH10), von Willebrand factor C domain containing 2 (VWC2), Spastic Paraplegia 20 (SPG20), Hepatocyte Nuclear Factor 4 (HNF4A), and cadherin 6 (CDH6) [59]. PEITC-treated androgen-dependent prostate cancer cells (LNCaP) showed growth attenuation and p53-independent cell cycle arrest through the up-regulation of p21^WAF1^ and p27. Mechanistic insights revealed that PEITC hyperacetylated histones, leading to decreased c-Myc expression and reduced c-Myc binding to Sp1, which in turn results in the decreased p21 repression [60].

PEITC inhibited the viability of A375 and Hs294T melanoma skin cancer cells. However, no significant toxicity of PEITC was reported on normal cell lines including HaCaT, A431, and VMM1 cells. PEITC effectively diminished HDAC1, HDAC2, HDAC4, and HDAC6 with a concomitant decrease in the expression of CBP and acetyl CBP/p300 and elevated expression of PCAF in A375 cells. Next, PEITC treatment led to the reduced expression of AcH4K12, AcH4K8, and AcH4K5, while a slight decrease was also observed in the expression levels of AcH3K14, AcH3K27, and AcH3K56 in melanoma cells [61]. Aberrant methylation at the CpG island of the glutathione S-transferase gene (GSTP1) promoter has been shown in prostate cancer, which could serve as a pathogenic marker. Sustained exposure of LNCaP cells to PEITC inhibited the expression and activity of HDAC1 and promoted de-methylation of the GSTP1 promoter region, which restored the activity of GSTP1 [62]. Another study investigated the possible epigenetic effects of PEITC on SET domain-containing lysine methyltransferase 7 (Setd7) that participates in the regulation of Nrf2. Exposure of PCa cells to PEITC resulted in a significant induction of Setd7 expression and elevated H3K4me1 enrichment in the promoter region of Nrf2 and GSST2 (glutathione S-transferase theta 2) [63]. PEITC suppressed the proliferation and induced apoptosis in SW480 colon cancer cells and attenuated mRNA expression of proinflammatory cytokines such as interleukin 8 (IL8), CCL2, and CXCL10 and mediated down-regulation of MMP7, STAT1, and NF-κB. Further investigations into the molecular mechanisms of PEITC revealed that its low concentrations increased the methylation of H3 at lysine 27 (H3K27) (which surrounds IL8 and CCL2 promoter regions) and caused hypermethylation at lysine 27 of Histone3 (H3K27) which surrounds the MMP7 promoter region [64].

The exposure of PC3 cells to PEITC led to the over-expression of miR-194, leading to bone morphogenetic protein1 (BMP1) down-regulation, and the study further suggested BMP1 as a direct target of miR-194. PEITC also promoted the suppression of the expression of MMP2 and MMP9 proteins, which in turn attenuated tumor invasion and cell migration in prostate cancer cells [65]. PEITC-treated PCa prostate cancer cells showed suppressed cell growth and proliferation through the decreased expression of PCA as a result of elevated expression of miR-17 [66]. Another study found that PEITC inhibited the proliferation and invasion ability of LNCaP cells by increasing the expression of miR-194, which is typically down-regulated in cancer cells. PEITC-mediated up-regulation of miR-194 was due to the reduced expression of MMP-2 and MMP-9 through targeting BMP1 in prostate cancer cells [67]. In vitro studies on organo-sulfur compounds indicated that PEITC inhibited proliferation, migration, and tumorigenicity and induced selective apoptosis in malignant glioma cells through caspase activation, the down-regulation of Bcl-2, STAT6, SMAD5, as well as the up-regulation of Bax and cytochrome-c. PEITC-mediated anticancer effects were due to ROS-dependent miR-135a up-regulation in the malignant glioma cells [66].

### 2.3. Diallyl Disulfide (DADS)

Diallyl disulfide was demonstrated to inhibit the activity of histone deacetylase by targeting multiple signaling cascades and inducing histone hyperacetylation to reactivate epigenetically silenced genes. Previously, DADS-exposed gastric cancer cells displayed elevated histone H3 and H4 acetylation and induced G_2_/M cell cycle arrest, which is accompanied by p21^WAF1^ up-regulation in HGC803 cells. Additionally, DADS exhibited antitumor effects in MGC803-xenografted nude mice, leading to G_2_/M cell cycle arrest and p21^WAF1^ up-regulation, which could be due to enhanced acetylation of histones H3 and H4 [68]. DADS-treated breast cancer cells demonstrated terminated DNA synthesis, increased sub-G_0_ populations, and the translocation of phosphatidylserine from the inner to the outer leaflet on the surface of the plasma membrane, indicating apoptosis induction. DADS-induced apoptosis was due to caspase-3 activation, Bcl-2 and Bcl-X_L_ down-regulation, as well as the up-regulation of Bax in MCF-7 cells, which possibly occurred in response to HDAC inhibition and the induction of H4 hyperacetylation by DADS [69]. DADS-treated Caco-2 and HT-29 colon cancer cells showed proliferation inhibition and cell cycle arrest at the G_2_ phase through increasing p21^WAF1/CIP1^ activity. A mechanistic approach revealed that DADS-mediated anti-proliferative effects were associated with the reduced HDAC activity as well as histone H4 hyperacetylation preferentially at lysine 12 and 16 (H4K12 and H4K16) [70]. Another interesting finding showed that DADS-mediated anti-proliferative effects and cell cycle arrest in Caco-2 and HT-29 could occur in response to the increased expression of CDKN1A mRNA and p21^WAF1^ as a result of elevated histone H3 and/or H4 acetylation in the promoter region of CDKN1A [71]. In benzo(a) pyrene (BaP)-exposed MCF-10A cells, DADS inhibited cell proliferation and peroxide formation, induced G2/M cell cycle arrest, and reduced DNA single-strand breaks [84].

DADS-treated MGC-803 gastric cancer cells showed reduced cell proliferation and invasion by down-regulating miR-222, which is typically over-expressed in gastric cancer, contributing to the gastric cancer metastases. Moreover, the miR-222 inhibitor and DADS combination further enhanced DADS-induced suppression of cell proliferation and invasion by TIMP3 down-regulation in MGC-803 cells [72]. Exposure of SGC-7901 gastric cancer cells to DADS significantly reduced cell viability and invasion and induced apoptosis through the up-regulation of miR-34a. The combination of DADS and miR-34a increased apoptosis induction in a synergistic manner while suppressing PI3K and Akt phosphorylation [73]. Another study demonstrated that DADS significantly reduced gastric cancer cell proliferation and induced apoptosis by targeting the Wnt signaling pathway through the up-regulation of miR-22 and miR200b. Furthermore, miR-22 and miR-200b were reported to synergistically suppress the growth and tumorigenicity in the DADS-treated animal model [74]. Previously, the anti-proliferative effects of DADS on breast cancer cells were investigated, and the results showed that DADS inhibited MDA-MB-231 cell proliferation, migration, and invasion and in vivo tumorigenicity by inhibiting Src, which consequently triggered the suppression of the SRC/Ras/ERK signaling pathway. DADS-mediated anti-proliferative effects on breast cancer were correlated with the elevated expression of miR-34a in MDA-MB-231 cells [75]. Diallyl disulfide (DADS) inhibited cell proliferation and the invasion of osteosarcoma by up-regulating miR-134 in U2OS, MG-63 cells, and in xenograft tumors. DADS-mediated miRNA over-expression led to the inhibition of forkhead box M1 (FOXM1), suppression of cyclin D1 and c-Myc, as well as the p21 up-regulation. Further investigations suggest the down-regulation of miR-134 reversed DADS-mediated anti-proliferative effects and induced over-expression of FOXM1, inhibiting the DADS-dependent suppression of proliferation and invasion in U2OS cells [76].

### 2.4. Benzyl Isothiocyanate (BITC)

Benzyl isothiocyanate reduced the cell viability of A375 and Hs294T in a dose- and time-dependent manner, whereas minimal BITC cytotoxicity was reported in normal cell lines (VMM1, HaCaT and A431). BITC treatment suppressed the expression of CBP, acetyl CBP/p300, HDAC1, HDAC2, HDAC4, and HDAC6, whereas PCAF was up-regulated in A375 cells. Furthermore, BITC-exposed malignant melanoma cells underwent the down-regulation of AcH4K5, AcH4K8, and AcH4K12 and reduced the acetylation of H3K56, H3K14,and H3K9 [61].

BITC treatment resulted in a significant up-regulation of miR-99a-5p, which further down-regulated insulin-like growth factor 1 receptor (IGFR1), mammalian target of rapamycin (mTOR), and fibroblast growth factor receptor 3 (FGFR3), leading to decreased cell viability and apoptosis induction in bladder cancer cells through the cleavage of poly (ADP-ribose) polymerase (cPARP) and pro-caspase-3 [77]. Another in vitro study indicated that the over-expression of miR-99a in BITC-treated bladder cancer cells occurred in response to the activation of the extracellular-signal-regulated kinase (ERK)/c-Jun signaling pathway [78]. BITC treatment attenuated cell proliferation by modulating the expression of micro RNAs such as miR-221 and miR-375, which are aberrantly expressed in pancreatic cancer cells. Pancreatic cancer cells transfected with miR-375 showed the down-regulation of insulin-like growth factor-binding protein-5 (IGFBP5) and caveolin-1 (CAV1), and BITC possibly targeted the up-regulation of IGFBP5 and CAV-1 through suppressing the expression of miR-221 and miR-375 [79].

### 2.5. Diallyl Thiosulfinate (Allicin)

Diallyl thiosulfinate (DATS) and dexamethasone co-treatment significantly reduced proliferation, colony, and sphere formation in multiple myeloma cells. Combinations of diallyl thiosulfinate and dexamethasone also induced apoptosis and G_1_/S cell cycle arresting in multiple myeloma cells. Further investigation revealed that the anticancer effects of diallyl thiosulfinate and dexamethasone co-treatment were due to the up-regulation of miR-127-3p and down-regulation of PI3K, p-mTOR, and p-Akt, resulting in the deactivation of the PI3K/AKT signaling pathway in multiple myeloma cells [85]. DATS-treated PANC-1 and GS799T pancreatic cancer cells displayed the suppression of proliferation, invasion, and migration and significant apoptosis induction by increasing E-cadherin expression and caspase activation and reducing the expression of vimentin and ZNF-689. Mechanistic insights revealed a significant up-regulation of miR-339-5p in response to DATS exposure to PANC-1 and GS799T cells [80]. The exposure of U251-MG and A172 cells to DATS led to the enhanced temozolomide sensitization and promoted apoptosis induction through the cappase-3 cleavage in glioblastoma cells that was comparatively higher than their single treatment. The enhanced temozolomide sensitivity to U251-MG and A172 cells and increased apoptosis induction was due to the DATS-mediated up-regulation of miR-486-3p, which directly targeted O6-methylguanine-DNA methyltransferase (MGMT) [86]. Furthermore, DATS exposure to AGS and HGC27 gastric cancer cells resulted in the decreased cell viability, migration, invasion, and apoptosis induction, which was linked with the alterations in the expression of miR-383-5p and ERBB4. Interestingly, DATS-mediated anti-proliferative effects occurred in response to miR-383-5p over-expression and down-regulation of ERBB4, which further led to Bax up-regulation, Bcl-2 down-regulation, and the reduced phosphorylation of PI3K and Akt [81].

## 3. Organo-Sulfur Compounds and Their Synergistic Effects on Cancer Cells 

A large number of studies have shown that combinatorial approaches to cancer treatment are effective. As cancer cells develop drug resistance, drug efficacy is reduced, and adverse drug reactions or severe side effects occur. The combination of organo-sulfur compounds with other phytochemicals or a standard cancer drug may synergistically reduce cell growth, proliferation, invasion, and migration. A growing body of studies suggests that this approach also contributes to apoptosis induction, autophagy, cell cycle arrest, and blockage of the DNA repair in cancer. In particular, the combination of a phytochemical with another phytochemical or a standard cancer drug targets cancer by increasing drug availability and therapeutic effects. Moreover, most of the organo-sulfur compounds tend to chemosensitize the cancer cells to standard cancer drugs. Combined doses of SFN and Withaferin A inhibited growth and cell proliferation and induced apoptosis in breast cancer cells. The molecular mechanism found responsible for the anti-proliferative potential of combined doses of SFN and Withaferin A on MDA-MB-231 and MCF-7 cells includes the decreased expression of Bcl-2, histone deacetylase (HDAC), DNMT1, DNMT3a, and DNMT3b, as well as the increased expression of bax [87]. In another study, the combination of SFN and Withaferin A blocked cell cycle progression from the S to G_2_ phase in breast cancer cell lines by down-regulating cyclin D1, cyclin dependent kinase 4 (CDK4), and pRB with a concomitant increase in p21 and E2F mRNA levels. Further mechanistic insights revealed the down-regulation of HDAC2 and HDAC3 and enhanced levels of H3K4Me3 (trimethylated lysine 4 of histone 3) in the promoter region of p21 in both breast cancer cell lines [88]. Furthermore, studies showed that genistein and SFN can act as epigenetic modulators. Particularly, genistein targets the inhibition of DNMTs, whereas SFN is a well-established HDAC inhibitor. Combined doses of genistein and SFN exerted more anti-proliferative effects and increased apoptosis induction in breast cancer cells as compared to their single treatments. Furthermore, MDA-MB-231 and MCF-7 treated with combined concentrations of genistein and SFN resulted in the decreased expression of HDAC2, HDAC3, KLF4, and hTERT, suggesting the alterations of epigenetic mechanisms in breast cancer cells [89]. Treating prostate cancer cells with a combined dose of SFN and diindolylmethane (DIM) led to the altered expression of genes including tumor suppressor genes, which was due to the alteration of DNA methylation. One such study has demonstrated that SFN combined with DIM significantly decreased the expression of DNMT1, DNMT3a, and DNMT3b in PC3, LnCAP, and PrEC cells, suggesting the de-methylation of the promoter region of cancer-associated genes [90]. CDKN2A, a tumor-suppressor gene silenced by DNA methylation, which has been reported hypermethylated and down-regulated in breast cancer cells. A combined dose of SFN and clofarabine increased CDKN2A mRNA expression moderately, while no relevant alterations in DNMT1 mRNA were recorded in invasive MDA-MB-231 cells. 5-aza-2′-deoxycytidine (5AZA) decreased CDKN2A methylation while increasing CDKN2A expression in MDA-MB-231 cells. When non-invasive MCF-7 cells were exposed to a combination of clofarabine and SFN, CDKN2A expression was tripled, and clofarabine alone caused CDKN2A de-methylation and a reduction in DNMT1 expression, while combinations of SFN and clofarabine were ineffective to de-methylate and prevent DNMT1 activity [91]. Furthermore, SFN caused cell growth inhibition and induced apoptosis in MCF7 and MDA-MB-231 cells with the IC_50_ values of 22 and 46 μM. SFN and clofarabine combinations further enhanced cell growth inhibition and apoptosis induction through the hypomethylation of PTEN and RARβ2, [92]. The exposure of PANC-1 pancreatic cancer cells to a combined dose of SFN and BITC displayed increased selective killing of cancer cells and the inhibition of STAT3 tyrosine phosphorylation compared to their individual treatments [93].

Furthermore, the synergistic anti-proliferative activity of PEITC and laccaic acid was investigated on colorectal cancer cells. Combinations of PEITC and laccaic acid suppressed cell viability and induced apoptosis in HT29 cells which was comparatively higher than their individual treatments. An in vivo study indicated that the inhibition of DNMT1, interleukin-6 (IL-6), and HDAC1 was apparent upon treatment with combined doses of PEITC and laccaic acid [94]. Combined doses of PEITC and paclitaxel were shown to exert synergistic anti-proliferative effects through apoptosis induction and cell cycle arrests in MCF-7 and MDA-MB-231 cells. The combined effect of PEITC and paclitaxel was found to be due to the reduced expression of CDK1 and Bcl-2 and the enhanced cleavage of PARP and bax up-regulation. Moreover, PEITC and paclitaxel combinations increased the acetylation of alpha tubulin by many folds in both cancer cell lines [95]. Individual doses of SFN, genistein (GE), and sodium butyrate (NaB) reduced breast cancer cell viability, which further increased when breast cancer cells were exposed to combined doses of SFN and GE, SFN and NaB, and SFN, GE, and NaB. The combinations also enhanced apoptosis induction and cell cycle arrest at G_2_/M phase in MCF7 and MDA-MB-231 cells. The mechanistic insights revealed the down-regulation of DNMT3A, DNMT3B, HDAC1, HDAC6, HDAC11, and histone methyltransferase upon treating cells with di-combination and tri-combination [96]. HT-29 AP-1 human colon carcinoma cells co-treated with SFN and EGCG showed enhanced cell survival inhibition in a synergistic manner wherein the low-dose combination decreased cell viability by up to 70%, while the high-dose combination inhibited cell viability by up to 40%. The mechanistic insights revealed the involvement of HDAC down-regulation, inhibition of cellular senescence, and SOD signaling [97]. Furthermore, the combination of β-phenylethyl isothiocyanate and vorinostat (HDAC inhibitor) significantly reduced the cell viability of HL60/LR cells (leukemia cell line) by inhibiting glutathione-associated enzymes that lead to glutathione depletion. Thus, the di-combination increased the chemosensitization of varinostat to leukemia cells and overcame HDAC inhibitor resistance through redox modulation [98].

## 4. Organo-Sulfur Compounds under Clinical Trials

To date, only a few organo-sulfur compounds have been reported to demonstrate significant chemopreventive efficacy in clinical studies against cancer. Previously, many clinical trials were conducted to evaluate the effects of organo-sulfur compound-rich cruciferous plant extracts, including broccoli, watercress, Brussels sprouts, and cabbage. Intriguingly, preclinical and clinical data show that consuming high levels of such cruciferous vegetables reduces the risk of cancer, owing to their bioactive organo-sulfur compounds, which mainly includes sulforaphane and phenethylisothiocyanate. For instance, in a pilot study, eight African American women aged between 21–50 years undergoing elective reduction mammoplasty were allowed to consume a broccoli sprout preparation containing 200 μM of sulforaphane. Further investigations showed that the dose was well tolerated when women administered broccoli sprout preparation an average fifty minutes prior to surgery, and the reported pharmacokinetic parameters included 0.92 ± 0.72 μM plasma levels of sulforaphane and 158.85 ± 93.89 μM urine levels of sulforaphane [99]. Another double-blind, randomized, and placebo-controlled study assessed the efficacy of sulforaphane in the recurrent prostate cancer patients with an elevated prostate-specific antigen (PSA). The clinical trial included a total of 78 patients, in which 38 were administered 60 mg of sulforaphane daily during the period of treatment (six months) and 40 were placebo. The log PSA slope in the sulforaphane-treated group was statistically lower in comparison to the placebo group, while the mean change in PSA levels was significantly lower in the sulforaphane-treated group than the placebo group during six months of treatment. The clinical study demonstrated excellent safety profiles with fewer gastrointestinal tract side effects in the patients administered with sulforaphane [100]. As per the website clinicaltrials.gov (accessed on 15 December 2022), a clinical trial (NCT01265953) used 30 mg of sulforaphane up to four weeks prior to prostate biopsies, and the key findings were HDAC inhibition and the expression of acetylated H3 and H4. The randomized, double-blind, placebo-controlled study (NCT00946309) included men with low- and intermediate-grade localized prostate cancer and was conducted up to phase II. Participants were administered broccoli sprout extract (100 μM of sulforaphane) for five weeks every other day; the study concluded that the consumption of high sulforaphane extract inhibits the growth of prostate cancer cells. Gastric cancer development is commonly correlated with Helicobacter pylori infection, and sulforaphane is a potent bactericidal isothiocyanate. Previously, 48 patients infected with *Helicobacter pylori* were allowed to consume broccoli sprouts containing 420 μM of sulforaphane precursor for a period of eight weeks, while the placebo group consumed alfalfa sprouts with no sulforaphane. Further investigations demonstrated that the consumption of sulforaphane-rich broccoli sprouts enhanced the chemoprotection of gastric mucosa against *Helicobacter pylori*-mediated oxidative stress by reducing its colonization [101]. In a phase II randomized, double-blind, placebo-controlled interventional study of 72 patients (NCT03232138), sulforaphane was investigated for chemoprevention in former smokers. Four sulforaphane tablets (each containing120 μM sulforaphane) were administered two times a day, while a placebo group consumed four tablets twice a day without sulforaphane for a period of twelve months. The purpose of the study was to investigate whether sulforaphane reverses some of the lung cancer cell morphology and improves the conditions of smokers who are at high risk of developing lung cancer. In a clinical trial (NCT01568996), broccoli-sprout-extract-enriched with sulforaphane was evaluated for its chemopreventive activity against atypical nevi-precursor lesions (assessment of STAT1 and STAT3 expression in melanoma). Eighteen participants were divided into three groups and allowed to consume broccoli sprout extract containing sulforaphane orally at doses of 50, 100, and 200 μM daily. In a clinical trial (NCT03182959), Avmacol^®^ tablets (broccoli sprout extract) were given to evaluate the effect on inner cheek cells, which may protect against toxins such as tobacco. The study hypothesized that broccoli sprout extract could activate the nuclear factor erythroid 2-related factor 2 (NRF2) signaling pathway in patients with tobacco-related head and neck squamous cell carcinoma.

Moreover, a phase I clinical trial (NCT00005883) was conducted to predict the efficacy and maximum tolerated dose of PEITC in lung cancer prevention in smokers. The patients (smokers) were allowed to consume phenethyl isothiocyanate orally four times per day for up to 30 days. Then, 3–6 patients received increasing doses of PEITC to determine the maximum tolerated dose, where 2out of 6patients experienced dose-limiting toxicities. Another clinical trial showed that PEITC is a potent inhibitor of the metabolic activation of NKK (tobacco carcinogen 4-(methylnitrosamino)-1-(3-pyridyl)-1-butanone) in smokers. Eighty-two smokers were included in the study, in which each subject consumed PEITC at a dose of 10 mg/mL of olive oil four times a day. Further analysis demonstrated that PEITC treatment decreased the NKK activation ratio by 7.7% in smokers, thereby targeting lung carcinogenesis [102]. Long-term effects secondary to cancer therapy in adults were assessed through a phase III randomized, longitudinal clinical trial (NCT02468882), which included two hundred participants who were allowed to consume watercress extract enriched with PEITC and other active organo-sulfur compounds. The study’s findings showed that 100 g of watercress extract supplementation per day during radiation therapy had a significant anticarcinogenic activity by suppressing carcinogen activation, cell cycle arrest progression, and apoptosis induction. A phase I and phase II clinical trial (NCT01790204) included 55 participants to study the chemopreventive potential of PEITC on oral cells with mutant p53. Each serving contains 55 gm of watercress juice in 220 mL of purified water containing PEITC and other organo-sulfur compounds. The findings suggested that the number of oral cells with mutant p53 was reduced after consuming PEITC derived from watercress juice. 

## 5. Organo-Sulfur Compounds: Sources, Chemical Structures, and Pharmacological Importance 

**Figure 4:**
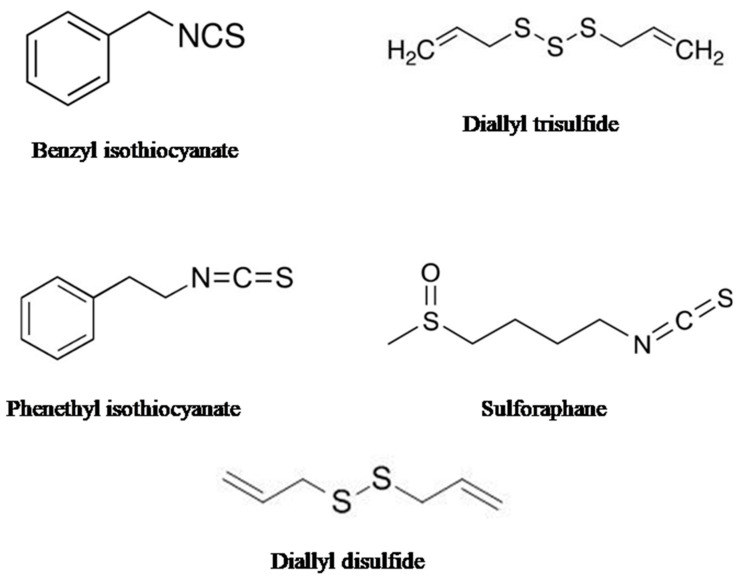
The figure shows the chemical structure of different organo-sulfur compounds.

It is well established that macro- and microelements play crucial roles in a plant’s growth and development and provide protection against the stress imposed by pathogens and the environment. Many of these elements also take part in the biosynthesis of enzymes as cofactors as well as participating in the biosynthesis of glucosinolates, leading to the development of defense mechanisms against pathogens. Among them, sulfur is one such element, necessary for the biosynthesis of many secondary metabolites, including phytoalexins as well as organo-sulfur compounds. The organo-sulfur compounds are mainly found in garlic (*Allium sativum*), onion (*Allium cepa*), cauliflower (*Brassica oleracea*, var. botrytis), broccoli (*Brassica oleracea* var. italica), watercress (*Nasturtium officinale*), Brussels sprouts (*Brassica oleracea*, var. gemmifera), and cabbage (*Brassica oleracea* var. capitata). Figure 4 shows the sources and chemical structures of organo-sulfur compounds.

*Allium sativum*, a member of the *Liliaceae* family and a native of Central Asia, is cultivated for its flavorful bulb. However, its flower, clove, and leaves have been reported to be used as traditional medicine, and recent research over the past decades has revealed the widespread pharmacological importance of *Allium sativum* and its organo-sulfur compounds, particularly allicin. In line with observations, in an in vitro study, allicin was shown to kill human lung pathogenic bacteria in clinical isolates of genera *Pseudomonas*, Streptococcus, and *Staphylococcus*, including multiple-drug resistant strains [103]. Another in vivo study showed that allicin in combination with vancomycin significantly reduced the formation of biofilm by *Staphylococcus epidermidis* in a rabbit model of prosthetic joint infection [104]. In an in vitro study, allicin was demonstrated to inhibit gram-negative *Pseudomonas aeruginosa* through the inhibition of bacterial surface adhesion, biofilm formation, production of virulence factors, and extracellular polymeric substance (EPS) secretion [105]. Similarly, another study has shown that allicin treatment inhibited biofilm formation and urease activity in a dose-dependent manner in twenty clinical isolates of *Proteus mirabilis*. However, allicin treatment did not show any inhibitory effects on hemolysin activity [106]. In addition to this, allicin was also reported to exert anti-tuberculosis effects in vitro and in vivo against both drug-sensitive and drug-resistant bacterial strains of tuberculosis (MDR and XDR). The mechanistic insights revealed allicin’s proinflammatory activities in macrophages, and in the murine model, allicin exhibited a strong protective Th1 response, which further led to the reduction in the mycobacterial load [107]. Furthermore, allicin’s effect against other diseases was also investigated. An in vitro study showed that allicin administration significantly reduced levels of serum creatinine, uric acid, urea, sodium, calcium, and phosphorus. Allicin administration resulted in a significant decrease in serum and renal levels of tumor necrosis factor and an increase in sodium oxide dismutase, glutathione peroxidase, and catalase levels in cisplatin-induced rats, suggesting allicin’s neuroprotective effects [108]. Apart from these actions, allicin has been shown to exert its antioxidant effects by inhibiting reactive oxygen species by down-regulating NADPH oxidizing enzymes and reducing ROS peroxidases [109]. Allicin has an excellent capacity to inhibit neuroinflammation by targeting TLR4/MyD88/NF-κB, P38, and JNK pathways [110]. In addition, allicin acts as a potent inhibitor of cholinesterase and butyrylcholinesterase, suggesting its anti-Alzheimer’s disease potential [111]. Another study showed that allicin can protect neurons from damage by decreasing inflammatory marker proteins and inducing apoptosis through the increased expression of Nrf2 (nuclear factor erythroid 2-related factor 2) [112]. 

SFN, PEITC, and BITC, organo-sulfur compounds, are mainly present in *Cruciferous* plants and have long been studied for their biological activities against different diseases, including cancer, bacterial infections, fungal infections, and neurodegenerative disorders. Interestingly, an intake of dietary PEITC and BITC has been shown to exhibit therapeutic and preventive effects against many diseases. Notably, PEITC and BITC act as antioxidant and anti-inflammatory agents, and they have also been demonstrated to exhibit antibacterial and anti-carcinogenic properties. A recent study demonstrated that PEITC and BITC had antibacterial activities against *Escherichia coli*. BITC exerted stronger antibacterial activity than PEITC, which was due to the disruption of surface morphology and a reduction in ATP levels in Shiga toxin-producing and enterotoxigenic *E. coli* [113]. Another in vitro and in vivo study showed that sulforaphane and PEITC inhibited bacterial growth and biofilm formation by *Vibrio cholerae*, which was due to bacterial cytoplasm shrinkage and the inhibition of the synthesis of genetic material as well as the suppression of activity of the virulence factors [114]. SFN and PEITC were investigated for whether these dietary organo-sulfur compounds can reduce the DNA adducts formation of aflatoxin B_1_ (AFB). The results of the study showed that SFN and PEITC reduced AFB-DNA adduct levels in some but not all hepatocytes, which was linked with the decreased expression of cytochrome P450 (CYP) 3A4 mRNA; thus, they suppressed aflatoxin-induced genotoxicity [115]. Moreover, PEITC and SFN were shown to exhibit neuroprotective effects through the suppression of ROS generation as well as the inhibition of matrix metalloproteinases by down-regulating extracellular-regulated protein kinase (ERK) activity, suggesting them as promising nutraceutical agents for the prevention and treatment of neurological disorders [116]. Previously, SFN and PEITC have been documented as cardio- and neuroprotective compounds, as they both can mitigate the adverse effects of ROS, inflammation, and apoptosis, thus protecting against cardiovascular diseases and neurodegenerative diseases [117]. SFN and PEITC were reported to exert anti-Parkinson’s disease effects by modulating the Nrf and MAPKs signaling pathways [118]. Intriguingly, SFN, PEITC, and BITC were assessed for their protective effects against oxidized low-density lipoprotein (LDL)-induced endothelial dysfunction. SFN, PEITC, and BITC exposure to HUVECs cells resulted in the activity of heme oxygenase (HO)-1, elevated glutathione levels, and antioxidant response element (ARE)-luciferase reporter activity as well as pre-treatment with SFN, PEITC, and BITC reversed oxidized LDL-induced ROS production and expression of ICAM-1, E-selectin, and VCAM-1 [119]. Several organo-sulfur compounds, including PEITC, SFN, and BITC, have been shown to exhibit antibacterial activities against many pathogenic and food spoilage bacteria, including species from genera *Escherichia*, *Bacillus*, *Klebsiella*, *Salmonella*, *Listeria*, and *Staphylococcus* [120]. 

Many lipid-soluble (γ -glutamyl-S-allylcysteine, γ -glutamyl-S-methylcysteine) and water-soluble (diallyl sulfide, diallyl disulfide, diallyl trisulfide, dipropyl sulfide, and dipropyl trisulfide) organosulfur compounds inhibited cholesterol synthesis in human and animal studies [121]. In vitro studies showed that DATS suppressed nitric oxide (NO) production and prostaglandin (PG)-E2 by down-regulating inducible NO synthase (iNOS) and cyclooxygenase 2 (COX-2) and exerted anti-inflammatory effects through the attenuation of tumor necrosis factor α and interleukin 1β in lipopolysaccharide-activated RAW 264.7 macrophages, which was linked with the suppression of TLR4 and NF-κB activity [122]. Furthermore, in vivo studies showed that DATS can act as a cardioprotective agent because DATS administration led to the protection of ischemic myocardium by the restoration of myocardial H_2_S and troponin I levels, reduced infarct size, and increased NO metabolites, owing to the activation of endothelial NO synthase [123]. Another in vivo study demonstrated the antioxidant and anti-inflammatory properties of DATS, in which DATS pre-administration (at a dose of 200 mg/kg body weight for 10 days) resulted in a significant attenuation of hepatotoxicity through improving hepatic functions and histopathological conditions by the amelioration of oxidative stress and inflammation in cyclophosphamide (CP)-induced hepatotoxicity in rats [124]. DATS pre-treatment at a dose of 10 μmol for 7 days resulted in the significant amelioration of dextran sulfate sodium-induced mouse colitis, presumably by down-regulating NF-κB and STAT3 inflammatory signaling [125]. Another in vivo study showed that diallyl disulfide (DADS) administration exerted anti-inflammatory activity either alone or in combination with indomethacin in cerulin-induced acute pancreatitis and associated lung injury by inhibiting the activity of serum amylase and myeloperoxidase as well as decreasing the expression of NF-κB and neurokinin-1-receptor (NK1R) [126]. DADS administration resulted in the antiedematous effects of carrageenan-mediated paw edemas in mice through its antioxidant and anti-inflammatory properties. The mechanistic insights revealed that DADS reduced the levels of C-reactive protein, IL-1β, IL-2, and TNF-α, as well as decreased activities of myeloperoxidase (MPO), COX-2, NO, PGE2, NF-κB, and monocyte chemoattractant protein-1 (MCP-1) [127]. 

## 6. Research Methodology 

Notably, we followed the guidelines of Preferred Reporting Items for Systematic Reviews and Meta-Analysis (PRISMA). In order to retrieve the published literature, we accessed online databases including Google Scholar, PubMed, Scopus, Science Direct, and Web of Science. We searched the literature on these authentic databases using keywords such as cancer epigenetics, cancer epigenome, role of miRNAs in cancer, HDACs, and DNMTs in cancer, epigenetic mechanisms of cancer, DNA methylation, and histone acetylation in cancer, epigenetic modulation of cancer through phytochemicals, SFN and cancer, PEITC and cancer, BITC and cancer, DADS and cancer, and allicin and cancer. Importantly, during searching, we excluded articles that were published in languages other than English. During our screening, we also excluded book chapters, case reports, editorials, conference abstracts, unpublished findings, and retracted papers. This review article includes and discusses in vitro, in vivo, and human studies performed on selected isothiocyanates for their plausible roles in the epigenetic modulation of cancer, which can be an alternative approach to epigenetic synthetic inhibitors of cancer. Data collected from each study considered for this review include information about the type of study (in vitro and in vivo), type of cancer, type of isothiocyanates, concentration of the isothiocyanate or plant extract, sources of isothiocyanates, and structure of the compounds. 

## 7. Conclusions and Future Directions

Extensive research on plants and their metabolites has revealed that they embrace distinct advantages, including novelty in terms of their diversity of compounds, cost-effectiveness, fewer side effects, and a low toxicity profile. In addition to these properties, plants and their bioactive compounds display enormous capacity to target various signaling pathways to mitigate cancer cell growth, proliferation, metastasis, angiogenesis, cancer stem cells (CSCs), cell survival, and the inhibition of cell cycle progression. Thereby, plant-derived bioactive compounds may serve as a potential source for developing drug candidates, including anticancer agents. A growing body of research indicates that organosulfur compounds have great potential for treating a wide range of infectious and non-infectious diseases, including cancer. In this review, we have compiled and analyzed the role of organo-sulfur compounds in modulating the expression of several oncogenic proteins and miRNAs that drive cancer initiation and progression at an epigenetic level. It is now a well-established fact that the majority of cancers initiate as the consequences of chronic inflammation, a deregulated cell cycle, and apoptotic signaling pathways. On the contrary, accumulating evidence indicates that organo-sulfur compounds also exhibit the ability to prevent carcinogenesis by exerting anti-inflammatory, antioxidant, anti-proliferative, anti-angiogenic, Ro-apoptotic, and cell cycle progression inhibitory effects. Therefore, these mechanisms of action of organo-sulfur compounds suggest that they can also be used as anti-carcinogenic and epigenetic modulators in cancer. Organo-sulfur compounds selectively target DNA methylation, histone modifications, and microRNA regulation, which may substantially help to combat cancer through epigenetic regulation. Additionally, several preclinical studies have shown the synergistic potential of organo-sulfur compounds in combination with another compound or a standard cancer drug. It is very important to mention that there is a significant knowledge gap in terms of clinical studies on epigenetic changes induced by organo-sulfur compounds in cancer cells. Further studies are needed to determine the most effective concentrations of organo-sulfur compounds because the concentrations reported in most of the preclinical studies remain pharmacologically irrelevant, despite the fact that sulforaphane and PEITC-enriched broccoli or watercress extracts have been registered at www.clinicaltrials.gov (accessed on 15 December 2022) by various researchers for cancer prevention and treatment. Secondly, there are certain limitations that need to be addressed, including poor bioavailability and biodistribution, rapid detoxification, low retention time in the blood, and target non-specificity. In conclusion, there are many challenges, such as the development of pharmacological formulations and delivery platforms, which may lead to increased bioavailability, slow removal, target specificity, and increased retention time of these organo-sulfur compounds.

## Figures and Tables

**Table 1:** The anti-proliferative effects and mechanism of action of organo-sulfur compounds against various type of cancers.

Organo-Sulfur Compound	Type of Cancer	Type of Study	Molecular Mechanism	References
SFN	Breast Cancer	In vitro	SFN caused significant improvement in the activities of p53, p21, and PTEN through epigenetic modulation of DNMT1, which resulted in the suppression of growth and proliferation of MCF-7 and MDA-MB-231 breast cancer cells.	[35]
SFN	Breast Cancer	In vitro	SFN inhibited hTERT activity through the down-regulation of DNMT1 and DNMT3, leading to the apoptosis induction in MCF-7 and MDA-MB-231 breast cancer cells.	[36]
SFN	Nasopharyngeal carcinoma	In vitroand in vivo	SFN inhibited tumor sphere formation and reduced the CSC-related proteins (SOX2 and ALDH). The mechanistic insights revealed restoration of WIF1 and down-regulation of DNMT1.	[37]
SFN	Prostate cancer	In vitro	SFN significantly decreased DNMT1 and DNMT3b expression in LNCaP prostate cancer cells, which were correlated with decreased methylation of cyclin D2 promoter regions containing c-Myc and Sp1 binding sites.	[38]
SFN	Liver cancer	In vitro	SFN reduced CPEB2 methylation and promoted the methylation of E2F3 and THAP1 along with the inhibition of HDAC5 and HDAC11, which led to decreased cell proliferation and apoptosis induction in HepG2 liver cancer cells.	[39]
SFN	Prostate cancer	In vitro	SFN treatment improved Nrf2 and NQO-1 activities in TRAMP C1 cells, which was linked with the reduced expression of HDACs DNMT1 and DNMT3a.	[40]
SFN	Cervical cancer	In vitro	SFN reverted the expression of CDH1, DAPK1, GSTP1, and RARβ, which was correlated with the decreased expression of DNMT3b and HDAC1.	[41]
SFN	Skin cancer	In vitro	SFN inhibited the expression of HDAC1, HDAC2, HDAC3, HDAC4, DNMT1, DNMT3a, and DNMT3b. SFN also augmented the expression of Nrf2, NQO-1, and HO-1 in skin cancer cells.	[42]
SFN	Prostate cancer	In vitro	SFN repressed the expression of hTERT in prostate cancer cell lines, which was linked with the acetylation of histone H3 lysine 18 and di-methylation of histone H3 lysine 4.	[43]
SFN	Breast cancer	In vitroand in vivo	SFN treatment led to the down-regulation of HDAC5 and repression of USF1 activity. Mechanistic insights also revealed elevated LSD1 ubiquitination in animals.	[44]
SFN	Lung cancer	In vitroand in vivo	SFN treatment resulted in the apoptosis induction and arrest at S-phase of the cell cycle in A549 and H1299 cells along with tumor growth inhibition in a mouse model, which was correlated with inhibition of HDAC activity and increased levels of acetylated histones H3 and H4.	[45]
SFN	Colon cancer	In vivo	SFN-rich diets reduced tumor burden in a mouse model, which was supposed to be due to the decreased expression of HDAC3 and augmented acetylation of histone H4.	[46]
SFN	Colon cancer	In vitro	SFN caused G/M cell cycle arrest in a time-dependent manner, which was further linked with the HDAC3 degradation.	[47]
SFN	Gastric cancer	In vitro	SFN treatment caused proliferation inhibition and apoptosis induction in AGS and MKN4 cells. Mechanistic insights revealed reduced expression of miR9 and miR-326, which further led to augmented levels of CDX1 and CDX2.	[48]
SFN	Lung cancer	In vitro	SFN treatment resulted in the increased expression of miR-9-3, which was due to the increased H3K4me1 enrichment and reduced CpG methylation in the miR-9-3 promoter. Further, they suggested attenuated expression of DNMT3a, HDAC1, HDAC3, and HDAC6.	[49]
SFN	Breast cancer	In vitro	SFN reduced the levels of DNMT1 and DNMT3b in MCF-7, MDA-MB-231, and SKBR3 cells. SFN also reduced the levels of miR23b, miR-92b, miR381, and miR-382.	[50]
SFN	Human colorectal cancer	In vitro	SFN down-regulated hTERT, HDAC1, and miR-21, which led to proliferation inhibition and apoptosis induction in HCT116 and RKO cells.	[51]
SFN	Non-small cell lung cancer	In vitro	SFN down-regulated miR-616-5p, vimentin, N-cadherin, and β-catenin and enhanced E-cadherin expression in 95D and H1299 cells.	[52]
SFN	Oral squamous cell carcinoma	In vitroand in vivo	SFN enhanced miR-200c expression and suppressed expression of ALDH1 and CD44.	[53]
SFN	Bladder cancer	In vitro	SFN increased expression of miR-200c, while it reduced ZEB1, COX-2, Snail, MMP-2, and MMP-9 in T24 cells.	[54]
SFN	Lung cancer	In vitro	SFN down-regulated miR19 and inhibited Wnt/β-catenin pathway activation in lung cancer cells.	[55]
SFN	Nasopharyngeal cancer	In vitro	SFN up-regulated miR-124-3p and decreased expression of STAT3, c-Myc, Oct-3/4 SOX2, Nanog, and β-catenin in HONE1 and SUN1 cells.	[56]
PEITC	Prostate cancer	In vitro	PEITC down-regulated DNMT3a, DNMT3b, HDAC1, HDAC2, HDAC4, and HDAC6 in LNCaP cells.	[57]
PEITC	Breast cancer	In vitro	PEITC decreased expression of DNMT1, DNMT3a, DNMT3b, HDAC1, and HDAC2 in breast cancer cells. PEITC also inhibited CSCs-related marker proteins including ALDH-1, Oct-4A, and SOX2.	[58]
PEITC	Colon cancer	In vitro	PEITC decreased methylation of PCDH10, VWC2, SPG20, HNF4A, and CDH6.	[59]
PEITC	Prostate cancer	In vitro	PEITC reduced c-Myc expression and up-regulated the expression of p21 and p27.	[60]
PEITC	Melanoma skin cancer	In vitro	PEITC diminished activities of HDAC1, HDAC2, HDAC4, and HDAC6 in A375 and Hs294T cells. PEITC treatment also reduced the expression of AcH4K12, AcH4K8, and AcH4K5 in melanoma skin cancer cells.	[61]
PEITC	Prostate cancer	In vitro	PEITC inhibited HDAC1 expression and promoted de-methylation at the promoter region of GSTP1 in LNCaP cells.	[62]
PEITC	Prostate cancer	In vitro	PEITC treatment improved Setd7 expression and enhanced H3K4me1 enrichment in PCa cells.	[63]
PEITC	Colon cancer	In vitro	PEITC at low doses increased the methylation of H3K27, leading to the attenuation of proinflammatory markers including IL8, CCL2, and CXCL10.	[64]
PEITC	Prostate cancer	In vitro	PEITC up-regulated miR-194, which led to the down-regulation of BMP1 in PC3 cells.	[64]
PEITC	Prostate cancer cells	In vitro	PEITC suppressed cell migration and invasion, which was due to the inhibition of MMP2 and MMP9.	[65]
PEITC	Prostate cancer	In vitro	PEITC suppressed cell growth and proliferation, which was linked with the increased expression of miR-17.	[66]
PEITC	Prostate cancer	In vitro	PEITC treatment increased miR194 expression, which resulted in the suppression of proliferation and invasion of LNCaP cells.	[67]
PEITC	Glioma	In vitro	PEITC up-regulated miR-135a and the down-regulation of anti-apoptotic proteins and caspase activation.	[66]
DADS	Gastric cancer	In vitroand in vivo	DADS augmented the acetylation of histones, H3, and H4 and up-regulated p21 in HGC803 cells and in mouse models as well.	[68]
DADS	Breast cancer	In vitro	DADS inhibited HDAC and caused the acetylation of histone H4 in MCF-7 cells.	[69]
DADS	Colon cancer	In vitro	DADS reduced HDAC activity and caused the acetylation of H4K12 and H4K27 in Caco2 and Ht-29 cells.	[70]
DADS	Colon cancer	In vitro	DADS caused the acetylation of histones, H3, and H4, which may lead to increased expression of CDKN1A at transcription level in Caco2 and HT-29 cells.	[71]
DADS	Gastric cancer	In vitro	DADS down-regulated miR-222, which further may result in the suppressed proliferation, invasion in MGC-803 cells.	[72]
DADS	Gastric cancer	In vitro	DADS up-regulated miR-34a, which may result in the reduction of cell viability and invasion and apoptosis induction in SGC-7901 cells.	[73]
DADS	Gastric cancer	In vitro	DADS caused miR-22 up-regulation, leading to reduced cell proliferation and apoptosis induction.	[74]
DADS	Breast cancer	In vitroand in vivo	DADS augmented miR-34a expression, resulting in the suppression of proliferation and metastasis in MDA-MB-231 cells and the inhibition of tumorigenicity in animal model through the attenuation of SRC/Ras/ERK signaling.	[75]
DADS	Osteosarcoma	In vitro and in vivo	DADS treatment significantly up-regulated miR-134, leading to the inhibition of proliferation and invasion in U2OS and MG-63 cells. DADS also exerted antitumor effects through the up-regulation of miR-134 in tumors.	[76]
BITC	Melanoma skin cancer	In vitro	BITC down-regulated AcH4K5, AcH4K8, and AcH4K12 and reduced acetylation of H3K56, H3K14, and H3K9 in A375 cells.	[61]
BITC	Bladder cancer	In vitro	BITC up-regulated miR-99a-5p, which was linked with the down-regulation of IGFR1, mTOR, FGFR3, and PARP cleavages.	[77]
BITC	Bladder cancer	In vitro	BITC treatment led to the over-expression of miR-99a, which may result in the activation of ERK/c-Jun signaling pathways.	[78]
BITC	Pancreatic cancer	In vitro	BITC suppressed the expression of miR-221 and miR-375, which may result in the proliferation of pancreatic cancer cells through the up-regulation of IGFBP5 and CAV-1.	[79]
DATS	Pancreatic cancer	In vitro	DATS treatment caused the up-regulation of miR-339-5p, which was possibly responsible for the inhibition of proliferation and metastasis and apoptosis induction in PANC-1 and GS799T cells.	[80]
DATS	Gastric cancer	In vitro	DATS over-expressed miR-383-5p and decreased cell viability, migration, and invasion of AGS and HGC27 cells.	[81]
